# MALINA: a web service for visual analytics of human gut microbiota whole-genome metagenomic reads

**DOI:** 10.1186/1751-0473-7-13

**Published:** 2012-12-07

**Authors:** Alexander V Tyakht, Anna S Popenko, Maxim S Belenikin, Ilya A Altukhov, Alexander V Pavlenko, Elena S Kostryukova, Oksana V Selezneva, Andrei K Larin, Irina Y Karpova, Dmitry G Alexeev

**Affiliations:** 1 Research Institute of Physico-Chemical Medicine of Russian Federal Medico-Biological Agency (RIPCM), Malaya Pirogovskaya 1a, Moscow, 119435, Russia; 2 Moscow Institute of Physics and Technology (MIPT), Institutskii per. 9, Dolgoprudny, Moscow Region, 141700, Russia; 3 Engelhardt Institute of Molecular Biology, Russian Academy of Sciences, Vavilov st. 32, Moscow, 119991, Russia

**Keywords:** Metagenomics, Human gut microbiota, Web-server, Statistical analysis, Visualization

## Abstract

MALINA is a web service for bioinformatic analysis of whole-genome metagenomic data obtained from human gut microbiota sequencing. As input data, it accepts metagenomic reads of various sequencing technologies, including long reads (such as Sanger and 454 sequencing) and next-generation (including SOLiD and Illumina). It is the first metagenomic web service that is capable of processing SOLiD color-space reads, to authors’ knowledge. The web service allows phylogenetic and functional profiling of metagenomic samples using coverage depth resulting from the alignment of the reads to the catalogue of reference sequences which are built into the pipeline and contain prevalent microbial genomes and genes of human gut microbiota. The obtained metagenomic composition vectors are processed by the statistical analysis and visualization module containing methods for clustering, dimension reduction and group comparison. Additionally, the MALINA database includes vectors of bacterial and functional composition for human gut microbiota samples from a large number of existing studies allowing their comparative analysis together with user samples, namely datasets from Russian Metagenome project, MetaHIT and Human Microbiome Project (downloaded from http://hmpdacc.org). MALINA is made freely available on the web at http://malina.metagenome.ru. The website is implemented in JavaScript (using Ext JS), Microsoft .NET Framework, MS SQL, Python, with all major browsers supported.

## Background

Whole-genome sequencing of environmental samples is producing data at an increasing pace. With the advent of high-throughput next-generation sequencing (NGS) technologies, a deeper insight into phylogenetic and functional composition of metagenomes has become feasible. The research community has a need for robust data analysis tools that allow efficient description of composition, classification and clustering coupled with comprehensive visualization of results, while providing means for comparative analysis within the context of all accumulated metagenomic data for same type of environment. There is a number of existing web services (including CAMERA [1], IMG/M [2], MG-RAST [3], METAGENassist [4] and others) and stand-alone applications (including QIIME [5] and SmashCommunity [6]) that integrate data visualization and statistical analysis functionalities with databases of publicly available metagenomic data, allowing the user to compare his/her own samples with those of other researches. However, the number of pre-loaded human gut metagenomic samples in the repertoire of these tools is limited.

The human gut microbiome is one of the most extensively studied subjects in metagenomic research. It is of particular interest to scientists because of its significant role in host health status. Representative reference genomes for many taxa have been sequenced, and a catalogue of prevalent gut microbial genes has already been established [7]. MALINA exploits this accumulated knowledge in the form of reference sequence sets to provide a means for analyzing human gut whole-genome reads within the context of world public metagenomic datasets. The inclusion of a vast set of existing human gut metagenomic datasets allows the user to check which datasets are most similar to his/her own data, and, if present, to examine the metadata of those and pose hypotheses based on similarities. Features of MALINA and existing software allowing human gut whole-genome metagenomic reads analysis are compared in Table 1.

**Table 1:** Comparison of MALINA and existing software allowing analysis of human gut metagenomic reads

**Name of software**	**Type**	**Number of included shotgun human gut microbiome samples**	**Supported input sequence formats**				**Statistical analysis**					**Visualization**				
			**454**	**Illumina**	**Sanger**	**SOLiD**	**Taxonomic profiling**	**Functional profiling**	**Taxa co-occurence analysis**	**Sample clustering**	**Group comparison**	**Abundance plots**	**PCA**	**BCA**	**MDS**	**Hierarchical clustering**
**CAMERA**	web-service	36	+	+	+	-	+	+	-	-	-	+	-	-	-	-
**IMG/M**	web-service	149	+	+	+	-	+	+	-	+	-	+	-	-	-	+
**MG-RAST**	web-service	13	+	+	+	-	+	+	-	+	+	+	+	-	-	+
**MALINA**	web-service	357	+	+	+	+	+	+	+	+	+	+	+	+	+	+
**METAGENassist**	web-service	39	-	-	-	-	-	-	+	+	+	+	+	-	+	+
**QIIME**	stand-alone	0	+	+	+	-	+	-	-	+	+	+	+	-	+	+
**SmashCommunity**	stand-alone	0	+	-	+	-	+	+	-	+	-	+	-	-	-	+

Note: although METAGENassist does not accept read sequences as input, it does accept precomputed feature matrices that can be obtained from reads of any of the mentioned sequencing technologies with the help of third-party software.

### Implementation

**Figure 1:**
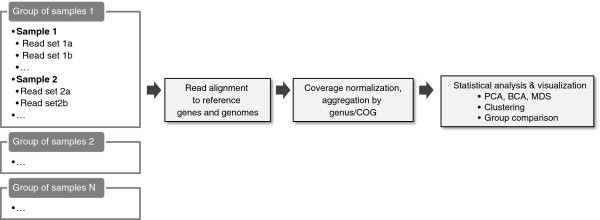
**MALINA workflow.** Input data and the main stages of analysis are illustrated.

The MALINA workflow is shown in Figure 1. As input, MALINA accepts short nucleotide reads of length starting with 35 bp. Color-space (SOLiD), as well as long (such as Sanger, 454) reads are supported. To our knowledge, MALINA is the first metagenomic analysis web-service supporting SOLiD color-space reads. It is beneficial, considering the increasing volume of metagenomic data sequenced using this technology. Files with reads are uploaded by FTP. Through the web interface, the user creates groups of samples, with each sample including one or more read sets. The files for a given sample are associated with appropriate read sets and prepared for analysis.

The MALINA analysis pipeline characterizes metagenomic composition in two ways: phylogenetic and functional - by assessing relative abundance of microbial genera and genes, correspondingly. In case of genes, total metabolic potential of all microbes is described. The quantitative profiling is based on alignment of reads to reference genomes and gene catalogue. The genome catalogue contains more than 440 genomes of human gastrointestinal bacteria obtained from HMP, NCBI and relevant studies of human gut microbiome. The gene catalogue of prevalent human gut microbial genes discovered by MetaHIT project consists of 3.3 million genes. After the reads are aligned to reference set, the resulting position-wise coverage of each sequence is normalized by its length and total number of reads in read set. Summed over genera (for genomes) or functional groups (clusters of orthologous groups, COGs [8]) for genes, it yields relative abundance of phylogenetic and functional units. For functional profiling, COG annotation from MetaHIT gene catalogue is used. Each metagenomic read set is thus described by two feature vectors.

**Figure 2:**
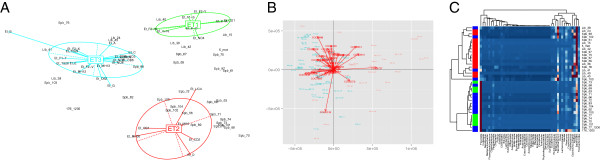
**Examples of visualizations by MALINA.** Metagenomic samples are denoted by text tags. (**A**) BCA plot of phylogenetic abundance, with the clusters visualized as ellipses and the samples connected with the centers of their respective clusters by lines. (**B**) PCA of COGs associated with vitamin metabolism, with drivers shown by red arrows. (**C**) Dendrogram heatmap of phylogenetic abundance based on hierarchical clustering algorithm (with samples as columns and genera as rows).

Feature vectors of the read sets selected by the user are subject to statistical analysis and visualization: boxplots of the most abundant genera/COGs, principal components analysis (PCA), clustering (partitioning around medoids [PAM] and hierarchical clustering) [9], multidimensional scaling (MDS) [10] and between-class analysis (BCA) [11] for the results of clustering. PCA plot shows 2D projection of feature vectors along the directions of maximum variance in the data, with arrows showing genera “drivers” that contribute most strongly to variation between samples. For COG groups, PCA plots are constructed separately for several functional classes: antibiotic resistance (COGs were collected from ARDB database [12]), transcription factors (COGs selected from total COG list according to description, i.e. “transcription regulator/factor/repressor”) and vitamin metabolism (COGs from KEGG [13] vitamin synthesis pathways). PAM clustering calculates the optimal number of clusters and assigns the samples to the clusters. BCA is a special case of PCA with respect to an instrumental variable (that is represented by cluster number here) producing plot that highlights differences between the clusters. The second implemented clustering algorithm, hierarchical clustering, produces dendrogram heatmap of abundance. Sample visualizations produced by MALINA are shown in Figure 2. Moreover, statistical analysis includes detection of genera and gene categories discriminatory among the clusters by the Mann–Whitney test [14] and Random Forests algorithm [15] as well as taxa co-occurrence analysis based on the abundance values correlation.

The plots can be downloaded as PDF files, and the relative abundance, clustering results and other output tables can be downloaded as tabulated text files. All features of MALINA are available without registration, via guest account. The user can register a dedicated account for free to provide privacy of uploaded data and results, as well as “analysis complete” notifications by e-mail.

An important functionality of MALINA is that besides the user’s own data, it is possible to co-analyze it with cohorts from large existing human gut metagenomic studies: 85 Illumina samples and 37 Sanger samples from MetaHIT study [7], as well as 139 Illumina samples from HMPDACC and 96 SOLiD samples from a new, previously unpublished Russian metagenomic study. Thus clustering functionality is of particular interest to researchers exploring human gut microbiota in relation to the concept of enterotypes [16] across a large number of samples. A stand-alone analysis of pre-loaded datasets is also available.

The user interface is implemented using Ext JS framework. Read alignment is performed using Bowtie [17]. In the interest of performance, MALINA does not filter reads using raw quality score, as the experience showed that filtration does not significantly increase the fraction of mapped reads. However, such preprocessing can be performed by the user manually. Coverage statistics are calculated using BEDtools [18]. Statistical analysis is implemented in R [19] using ade4 [11], cluster [20], ecodist [21], fpc [22] and randomForest [23] packages. The pipeline steps are integrated using Oracle database, Microsoft .NET framework and Python.

## Conclusions

MALINA allows an easy and intuitive way to infer metagenomic composition from reads and to analyze similarity of samples and organization into clusters within the global context of human gut metagenomic datasets. The features include statistical analysis methods like clustering and group comparison, as well as illustrative visualizations of phylogenetic and functional composition. The support for color-space SOLiD reads is a unique feature that makes MALINA a particularly valuable service to the growing community of researchers using SOLiD technology for metagenomic analysis.

The reference gene catalogue used in MALINA will be updated regularly as new version of MetaHIT data becomes available. In the future, it is planned that additional detailed metadata will be associated with the samples, allowing the user to check if newly sequenced samples are similar to certain groups distinguished by medical, ethno-geographic or dietary factors. The further development of the web service will include updates of the human gut samples database from Russian population as well as from other new studies. Support for diverse types of environment profiles besides human gut and additional methods for statistical analysis and visualization will be added.

### Availability and requirements

Project name: MALINA

Project page: http://malina.metagenome.ru

Operating system: platform independent web site

Programming languages: Microsoft C# .NET, JavaScript, R, Python

Other requirements: None

License: FreeBSD

Any restrictions to use by non-academics: None

## Abbreviations

BCA: Between-Class Analysis; HMPDACC: Human Microbiome Project Data Acquisition and Coordination Center; MDS: Multidimensional scaling; PCA: Principal Components Analysis.

## Competing interests

The authors declare that they have no competing interests.

## Authors’ contributions

ESK, OVS, AKL and IYK extracted metagenomic DNA, prepared the libraries for sequencing and performed sequencing on SOLiD 4, yielding readsets used as part of web-service. DGA, AVT and MSB designed the pipeline, performed coding and processed sequenced data. IAA designed the database and developed Web-interface. ASP maintained the database, developed statistical analysis and visualization module. AVT, AVP and ASP wrote the manuscript text. All authors read and approved the final manuscript.

## Funding

This work was supported by State Contracts 16.512.11.2111, 16.552.11.7034 and RFBR Grant 12-07-90008.
